# Trend Analysis and Predictions of Coronavirus Disease 2019 in Ethiopia

**DOI:** 10.34172/jrhs.2021.59

**Published:** 2021-08-12

**Authors:** Abiyot Negash Terefe, Samuel Getachew Zewudie

**Affiliations:** ^1^Jimma University, College of Natural Sciences, Department of Statistics, Jimma, Oromia, Ethiopia; ^2^Mizan-Tepi University, College of Natural and Computational Sciences, Department of Biology, Tepi, Ethiopia

**Keywords:** ARIMA, COVID-19, Ethiopia, Prediction, Trend

## Abstract

**Background:** Coronavirus Disease 2019 (COVID-19) is affecting both lives of millions of people and the global economy of the world day by day. This study aimed to determine the trend of COVID-19 and its predictions in Ethiopia.

**Study Design:** This study was conducted based on a time series design.

**Methods:** The required data were collected from the Ethiopian COVID-19 monitoring platform beginning from the onset of the disease in the country until March 28, 2021. Furthermore, the auto-regressive integrated moving average models were used on daily-based time series. The Poisson and Negative Binomial regression were also employed to notice the effects of months on the transmission and disease-related human deaths.

**Results:** The mean daily infection and death of COVID-19 in Ethiopia were 533.47±466.62 and 7.45±6.72, respectively. The peaks of infection and deaths in this country were in March, 2021, and August, 2020. In addition, the trend of daily new deaths (*P*=0.000) and infection (*P*=0.000) was significantly increasing. It is expected that around 10 million (8.6%) and 138,084.64 (0.12%) Ethiopians will be infected and die, respectively.

**Conclusions:** The disease transmission and deaths vary from day to day and month to month. The highest peaks of COVID-19 infection and death were in March 2021 and August 2020. For the next end of August 2021, the COVID-19 daily new infection, new death, total case, and total death are expected to be increased. If this epidemic disease is not controlled, Ethiopia will face a severe shortage of hospitals, and the outbreak even becomes worse.

## Introduction

Coronavirus Disease 2019 (COVID-19) pandemic is affecting the lives of millions of people and the global economy of the world day by day. Globally, the rate of infections and deaths are rising, and the evidence of local transmission of the disease has been found in many countries across all six World Health Organization (WHO) regions. From the onset of the disease in December 2019, more than 2,833,079 cases of COVID-19 have been reported in more than 200 countries and territories resulting in over 197,354 deaths^1^. This probable increment of cases has a tremendous effect on health resources that are shifted to this deadly virus response, and the healthcare system becomes overwhelmed with COVID-19 cases^2^. Therefore, it is vital to control the spread of the virus at the earliest stage^3,4^.

Recent reports from developing countries of Africa are indicating the increasing rates of new infections and deaths with the worst scenario in countries of sub-Saharan, including Ethiopia, where the health facilities are less furnished. The total number of COVID-19 cases in Africa is reported as 4,219,608 cases, while it is 200,563 in Ethiopia^1^. In Ethiopia, all national regional states are affected by a disease with a higher spread in its capital city, Addis Ababa. The COVID-19 pandemic is likely to have adverse effects on the key sectors of the Ethiopian economy^5^. People afflicted with the disease deny care due to poor understanding, and they seek care due to the fear of stigma^6^.

 Even though Ethiopia has made several measurements to prevent and control the pandemic disease, new cases and deaths are still increasing. The pandemic has the potential for superior loss of lives in the country. Many Ethiopians live in crowded conditions, and they do not even mask up. Therefore, the spread of the disease and deaths are escalating. Ethiopia has not projected a peak in cases and deaths for a longer time and months in which the number of COVID-19 cases and deaths reaching its climax has not been predicted yet. However, accurate predictions of infections and deaths help the national government prepare policies to control the spread of the infection. It also helps develop plans for the concerned body long-term trend of the COVID-19 epidemic to determine the transmission characteristics of the virus and design proper prevention and control mechanisms (guidelines) beforehand. Therefore, this study aimed to determine the trend of COVID-19 and its predictions in Ethiopia.

## Methods

###  Study Area and Population

This time series study was conducted in Ethiopia, which is situated in the eastern part of Africa with its capital city, Addis Ababa (at 2,300m [7,500 ft] above sea level). The country is administratively divided into 10 regions (e.g., Tigray, Afar, Amhara, Oromia, Benishangul-Gumuz, Gambela, Ethio-Somali, SNNPR, Harari, and Sidama) and two administrative towns, including Addis Ababa and Dire Dawa with a total population of 115 million according to the report of Worldometer^1^. The altitude and climate of the country are mostly variable, and its climate goes from hot to arid lowlands to the cool of the plateau. The altitude of its different districts of the city ranges from 2,100 to 2,700m (7,000 to 9,000 ft) with a mild climate. Nights are cold from November to February, and they reach below 10°C (50°F), while days are pleasantly warm (23/25°C [73/77°F]), except for July and August, at the height of the rainy season when they drop to about 20°C (68°F). The period from March to May, as often happens in the country, is the warmest of the year^7^.

###  Data Collection

 The data were collected from the Ethiopian COVID-19 monitoring platform (the daily news of the country health report) beginning from the onset (March 13, 2020) of the disease until March 28, 2021. The variables considered during data collection were daily new deaths, new cases, total recovery, total cases, and months that were collected from the platform.

###  Operational Definition

 The COVID-19 positive confirmed case is an individual whose viral confirmatory test is performed using throats/nasal swab or saliva test and whose specimen is tested positive for SARS-CoV-2, which is the virus that causes COVID-19.

###  Statistical Methods

Different statistical models were used to predict the incidence and transmission patterns of COVID-19 pandemic disease. Auto-regressive integrated moving average (ARIMA) models were also utilized on daily-based time series. The model adequacy was checked using the autocorrelation function and partial autocorrelation function (PACF) of the residuals and the Ljung-Box Q test. The general linear model of the Poisson and negative binomial (NB) regression were also employed in this study. The variance of the random variable is constrained to equal the mean, and researchers routinely employ more general specifications, usually, the NB model, which is the standard choice for a basic count data model^8^. The data were analyzed in R software (version 4.0.4), and a p-value less than 0.05 was considered statistically significant.

## Results

 Out of 115 million Ethiopians, 2,332,735 cases were tested for COVID-19 from March 13, 2020, to March 28, 2021. Of these, 200,564 individuals had COVID-19, while 2801 cases died. The mean±SD of the daily infection was obtained at 533.47±466.62, and high variations in the daily transmission of the disease were recorded during this period. The maximum daily test of COVID-19 was 25,158. Moreover, the maximum daily death due to this pandemic was determined at 30 cases (7.45±6.72). However, the maximum daily recovery of the COVID-19 was obtained at 4,493 cases (385.11±474.62).

In the country, the highest peaks of infections and deaths were recorded in March2021 and August 2020. Comparatively, the value of March is found low due to the number of cases, and deaths rate in 2020 was low, and this has pulled down below the data of August 2020 (Table 1).

**Table 1:** Descriptive results of COVID-19 epidemic disease in Ethiopia for the last March 28, 2021

**Months**	**No. of daily cases**	**Daily new cases**		**Daily new deaths**	
		**Mean**	**SD**	**Mean**	**SD**
April	30	3.50	2.60	0.10	0.40
August	31	1116.16	448.52	17.26	5.80
December	31	457.74	93.28	7.00	3.92
February	28	765.07	143.22	9.71	4.25
January	31	431.81	128.65	5.48	4.19
July	31	370.39	249.72	5.39	4.43
June	30	163.43	65.96	3.23	2.42
March	42	988.55	777.78	10.38	9.58
May	31	33.32	37.04	0.26	0.63
November	30	463.50	99.12	7.90	3.39
October	31	671.00	160.61	8.74	3.82
September	30	774.57	234.21	12.97	4.87

###  Linear Trend model for the Daily transmission and deaths of COVID-19

The linear trend models in this study were conducted based on the daily new cases and deaths, while the prediction of the models was carried out based on the daily new cases, new deaths, total cases, and total deaths of COVID-19. The model predictions were calculated for the coming end of August 2021. The estimated time series trend for the daily new death was 0.03 (*P*=0.000), and this indicates its significantly increasing trend. Moreover, the estimated coefficient for the daily new cases was 2.66 (*P*=0.000), indicating a significantly increasing trend nearly by 3 (on average) on a daily basis. The estimated coefficients for the daily new infections and deaths in March 2021 were 43.87 (*P*=0.000) and 0.48 (*P*=0.004), respectively, indicating a significant increase in transmissions and deaths nearly (in average) by 44 and 0.48 on a daily basis, respectively. In August 2020, the transmission of COVID-19 new cases was increased nearly by 40 (*P*=0.000) daily on average.

 From this, March 2021 was pulled down because of March 2020. The highest peaks of the transmission and deaths regarding month were in March 2021 and August 2020.

###  Time-series Plots of daily transmissions and deaths of COVID-19

**Figure 1:**
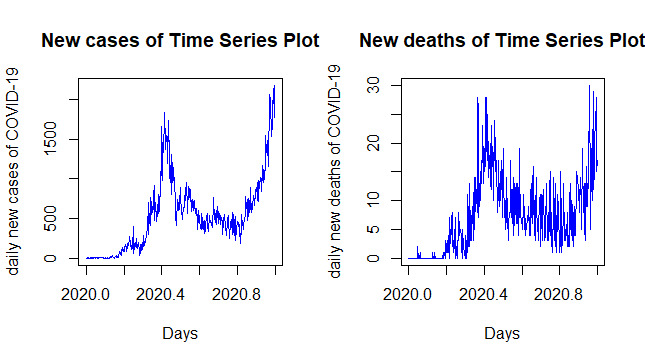
Time series plots of daily new cases and new deaths of COVID-19 in Ethiopia

The plots of COVID-19 daily new cases and deaths showed an upward trend from March 13 2020 to August 2020; a downward trend from August 2020 to January 2021; and an upward trend from January 2020 to March 28, 2021 (Figure 1). The consecutive observations of COVID-19 epidemic disease revealed no steady increase or decrease over 376 days. There was no identical pattern of trends of daily COVID-19 new cases and deaths in each month, as the relative amplitude of seasonal changes were remained not constant and not stationary through the period (Figure 1).

Since the Augmented Dickey-Fuller Tests (P=0.960 and *P*=0.7144) were more than 0.05, the data were transformed and differed for the stationary (Figure S1), and the Augmented Dickey-Fuller Tests (*P*=0.010 and *P*=0.010) were recorded as less than 0.05. Following that, the data were found fulfilling the stationary criteria after they differed. Moreover, the Shapiro-Wilk normality test and normal Q-Q plot showed the daily new cases (*P*=0.057) and deaths (*P*=0.083) and fulfilled the normality assumption after the data differed.

 Except for lag 1 and 2 auto-correlations and the first six lags for PACF, the other lags do not exceed the significant bounds, which was more or less good for the daily new cases. Moreover, except for lag 1 and 2 auto-correlations and lag 1 for the PACF, the other lags do not exceed the significant bounds and that is more or less good for the daily new deaths model (Figure S2). According to the Ljung-Box tests, there were no significant autocorrelations between successive forecasting errors. The Ljung-Box tests yielded a p-value of more than 0.05 indicating that the models were free from serial correlation. The values were normal as they rest on a line and were not all over the place. All the models support the assumption that there was no pattern in the residuals, and therefore, the model residuals were homoscedastic, and the prediction of the models was appropriate.

In this finding, both daily new cases and new deaths data were stationary (Augmented Dickey-Fuller Tests, *P*=0.010) time series. The results showed that the models that best fit daily new cases and new deaths were ARIMA (0, 0, 1) and ARIMA (2, 0, 1), respectively. The confidence interval (CI) for the coefficient of models parameters of both daily new cases and new deaths does not include zero, indicating significant parameter, except for AR1 (𝞿) for daily new deaths (Table 2).

**Table 2:** Best Parametric estimates of the ARIMA models

	**ARIMA(0, 0, 1) model for daily new case**				**ARIMA(2, 0, 1) model for daily new death**			
**Parameters**	**Estimate**	**SE**	**z- cal**	**95% CI**	**Estimate**	**SE**	**z- cal**	**95% CI**
AR1(𝞿)	--	--	--	--	0.05	0.06	0.73	-0.08, 0.17
AR2(𝞿)	--	--	--	--	-0.14	0.06	-2.44	-0.26, -0.03
MA1(𝞱)	-0.52	0.04	-12.33	-0.60, -0.44	-0.78	0.04	-19.82	-0.86, -0.71

 The models for COVID-19 disease daily new cases and deaths in Ethiopia were as follows:

 The substituted estimates of COVID-19 daily new cases:


(1) $$X_{t}=-0.5183Z_{t-1}+\varepsilon_{t}$$

The substituted estimates of COVID-19 daily new deaths:


(2) $$X_{t}=0.0456X_{t-1}-0.142X_{t-2}-0.782Z_{t-1}+\varepsilon_{t}$$

###  Prediction for COVID-19 cases

**Figure 2:**
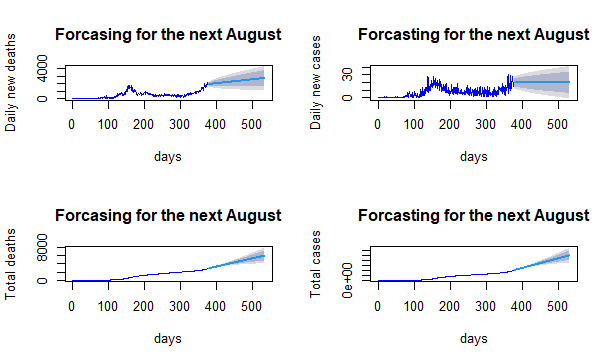
Ethiopian COVID-19 daily forecasts for the end of August 2021

Forecasts based on ARIMA models for the next end of August regarding COVID-19 daily new infection, new death, total cases, and total deaths are expected to be 2739.49, 19.62, 503,835.70, and 5862.07, respectively, showing an increase at an alarming rate (Figure 2). Therefore, Ethiopia should work to control and prevent the disease timely to solve the nightmare of the people and shortage of hospitals and facilities in the future.

###  Generalized Linear Regression Models for COVID-19 daily transmission and deaths

 Of 115 million Ethiopians, only 2,332,735 (2.03%) people were enrolled in COVID-19 tests, indicating the low capacity of daily testing in the country. Of these tested people, 200,564 (8.6%) cases were confirmed as positive for COVID-19, and 2801 (0.12%) of them died by the pandemic. Based on this, if testing of all 115 million Ethiopians were carried out, 9,887,475.75 would be infected and 138,084.64 of the people would die of the disease.

The mean and variance for the COVID-19 daily new death were obtained at 7.45 and 45.21, respectively, with the variance to mean ratio of 6.07. Moreover, the mean and variance for the daily new cases were 533.47 and 217,734.68, respectively, with the variance to mean ratio of 408.15. The Poisson model is often criticized for its restrictive property that the conditional variance equals the conditional mean; however, the real-life data are often characterized by overdispersion that the variance exceeds the mean. The NB regression model is a generalization of the Poisson regression model that allows for overdispersion by introducing an unobserved heterogeneity term for observation. For this, the NB model is recommended to model the data. The Akaike Information Criterion (AIC) and Bayesian Information Criteria (BIC) for the daily new deaths and daily new cases of NB models are less than those in the Poisson regression models, which implies that NB models are the best to account for the dispersion of daily new deaths and daily infection of COVID-19 disease (Table 3).

In the NB regression model, except for May, all months are significantly associated with the daily new deaths. In addition, August was 5.15 times more likely to have higher COVID-19 associated deaths, compared to April. All months were significantly associated with daily new deaths of COVID-19, which were more likely to have higher deaths than April. In the NB regression model, all months are significantly associated with the daily new cases. Furthermore, all months in a year are found to have a more likely higher rate of daily new infections, compared to April (Table 3).

 To compare those models in a statistical sense using the AIC and BIC for the daily new cases and new deaths, the model with the smallest AIC and BIC value is considered the best one. Therefore, for both daily new cases and new deaths, the NB regression model is responsible to account for the data (Table 3).

**Table 3:** Poisson and negative binomial regression for the daily new deaths and new cases of COVID-19 and model comparison

	**Daily New deaths**				**Daily New cases**			
	**Poisson regression**		**Negative Binomial**		**Poisson regression**		**Negative Binomial**	
**Parameters **	**Estimates (SE)**	***P*** **-value**	**Estimates (SE)**	***P*** **-value**	**Estimates (SE)**	***P*** **-value**	**Estimates (SE)**	***P*** **-value**
(Intercept)	-2.3 (0.58)	0.000	-2.3 (0.59)	0.000	1.25 (.10)	0.000	1.25 (0.17)	0.000
April	Ref.		Ref.		Ref.		Ref.	
August	5.15 (0.58)	0.000	5.15 (0.60)	0.000	5.77 (0.10)	0.000	5.77 (0.22)	0.000
December	4.25 (0.58)	0.000	4.25 (0.60)	0.000	4.87 (0.10)	0.000	4.87 (0.22)	0.000
February	4.58 (0.58)	0.000	4.58 (0.60)	0.000	5.39 (0.10)	0.000	5.39 (0.22)	0.000
January	4.00 (0.58)	0.000	4.00 (0.60)	0.000	4.82 (0.10)	0.000	4.82 (0.22)	0.000
July	3.99 (0.58)	0.000	3.99 (0.60)	0.000	4.66 (0.10)	0.000	4.66 (0.22)	0.000
June	3.48 (0.59)	0.000	3.48 (0.60)	0.000	3.84 (0.10)	0.000	3.84 (0.22)	0.000
March	4.64 (0.58)	0.000	4.64 (0.59)	0.000	5.64 (0.10)	0.000	5.64 (0.21)	0.000
May	0.95 (0.68)	0.000	0.95 (0.69)	0.170	2.25 (0.10)	0.000	2.25 (0.22)	0.000
November	4.37 (0.58)	0.000	4.37 (0.60)	0.000	4.89 (0.10)	0.000	4.89 (0.22)	0.000
October	4.47 (0.58)	0.000	4.47 (0.60)	0.000	5.26 (0.10)	0.000	5.26 (0.22)	0.000
September	4.87 (0.58)	0.000	4.87 (0.60)	0.000	5.40 (0.10)	0.000	5.40 (0.22)	0.000
AIC	2268.50		1968.50		57889.00		5011.00	
BIC	2315.69		2019.56		57935.86		5062.08	

###  Model Adequacy checking

 For a Poisson distribution, a low agreement was found between the expected and observed counts with 0 up to 3, 8, 9, and 11 counts that were under-predicted, while counts 4 and 6 were over-predicted. The Rootogram for the NB model shows much better agreement, compared to the Poisson model. Departures from the expected counts are much smaller, and the count model is much better fitted. Some small deviations from the observed data remain; however, that is to be expected (Figure S3).

## Discussion

 Globally, several studies were conducted that reported variable highest peak time of infections across countries. In Ethiopia, the highest rates of infections were recorded in August 2020 and March 2021, and this is consistent with the results of a study conducted in China on the prediction for the development of COVID19 in global major epidemic areas through empirical trends utilizing state transition matrix model.

Similarly, the highest peaks in South Korea, Italy, and Iran were from March 6 to 12, March 10 to 24, and March 10 to 24, respectively^9^. In contrast to this, a study from Iran revealed increases in the number of total cases in April 2020, whereas a study in Turkey reported a decrease in the total number of COVID-19 confirmed cases in March 2020^10^. Moreover, in India, the number of total cases and daily new deaths have been found to increase in April 2020^11^.

On the other hand, the USA expected the highest peak of infection in July 2022, while it was May 2021 in India. Russia and Brazil have also witnessed the peak of infection in May 2020 and July 2020 respectively, while it was in August 2020, in Peru and Colombia^12^.

In Africa, a similar prediction was conducted and predicted the fall of cases in August 2020^13^, which was inconsistent with the results of the current study in which the highest peak of infection was in August 2020. This could be due to differences in the transmission of this pandemic and the controlling efforts across countries.

A study was performed in Bangladesh on the prediction of the epidemic trend of COVID-19, and the results showed an increase in the number of tests with an amplified number of new positive cases^14^, which was consistent with the findings of the current study.

According to this study, out of COVID-19 tested individuals, 8.6% of them were infected by the disease, and 0.12% of them died due to this pandemic. Moreover, it is predicted that 9,887,475.75 cases would be infected, and 138,084.64 individuals would die of the disease if all Ethiopians are tested. Therefore, the transmission nature of the pandemic is escalating, and this is in line with the results of a study carried out in Ethiopia to assess how the Ethiopian government has executed administrative actions and managed risk communications and community engagement. The transmission of the disease could be escalated due to low administrative actions, low management of risks, and low community engagements to control the epidemic^15^.

 This study revealed the prediction for the end of August 2021, and the numbers of cases are expected to be high; additionally, the trend for the cases and deaths will be expected to increase. This is consistent with the findings of a study performed nine months ago in Ethiopia on COVID-19 prediction. The results revealed that Ethiopia would face many hospital shortages and quarantine places, and this justified designing strategies as there may be a shortage of hospitals starting from the next three months.

## Limitations

 This study only contains months as the variable that would influence the COVID-19 new cases and deaths. Moreover, the exclusion of the nutritional, cultural, and environmental factors were other limitations of the study.

## Conclusion

 The disease transmission and associated deaths vary from day to day and month to month. The trend of daily transmission of the cases and deaths was increasing in Ethiopia. The peak of COVID-19 infection and death were observed in March 2021 and August 2020. The pandemic disease may be manageable if all concerned bodies of the country engage in aggressive implementation plans, advice of WHO, and the country’s guidelines related to this pandemic. Based on the current prediction of the future COVID-19 transmission, the Ethiopian Ministry of Health can frame policy decisions and actions in order to control the spread. If not, in the coming two to three months, Ethiopia will have a higher possibility of facing severe transmission. The outbreak even becomes worse, followed by shortages of hospitals and facilities for the treatment.

## Acknowledgments

 Not applicable.

## Conflict of interests

 The authors declare no conflict of interest.

## Funding

 Not applicable.

## Highlights

- The trend for the daily new deaths and cases was found significantly increasing.
- The highest peaks for transmission and deaths were recorded in March 2021 and August 2020.
- At the end of August 2021, daily new infections, new deaths, total cases, and total deaths for Coronavirus Disease 2019 (COVID-19) are expected to increase alarmingly.
- It is expected that about 10 million (8.6%) Ethiopians will be infected by the disease.
- The negative binomial model was the best to account for the dispersion of daily new deaths and daily infections of the COVID-19 pandemic.
